# The *AP2/ERF* Gene Family in *Triticum durum*: Genome-Wide Identification and Expression Analysis under Drought and Salinity Stresses

**DOI:** 10.3390/genes11121464

**Published:** 2020-12-07

**Authors:** Sahar Faraji, Ertugrul Filiz, Seyed Kamal Kazemitabar, Alessandro Vannozzi, Fabio Palumbo, Gianni Barcaccia, Parviz Heidari

**Affiliations:** 1 Department of Plant Breeding, Faculty of Crop Sciences, Sari Agricultural Sciences and Natural Resources University (SANRU), Sari 4818166996, Iran; sahar.faraji@rocketmail.com (S.F.); sdklkr@ymail.com (S.K.K.); 2 Department of Crop and Animal Production, Cilimli Vocational School, Duzce University, Duzce 81750, Turkey; ertugrulfiliz@gmail.com; 3 Laboratory of Genomics for Breeding, DAFNAE, Campus of Agripolis, University of Padova, Legnaro, 35020 Padova, Italy; alessandro.vannozzi@unipd.it (A.V.); fabio.palumbo@unipd.it (F.P.); gianni.barcaccia@unipd.it (G.B.); 4 Faculty of Agriculture, Shahrood University of Technology, Shahrood 3619995161, Iran

**Keywords:** *AP2/ERF* gene family, *Triticum durum*, gene expression, genome sequence, abiotic stresses

## Abstract

Members of the *AP2*/*ERF* transcription factor family play critical roles in plant development, biosynthesis of key metabolites, and stress response. A detailed study was performed to identify *TtAP2*s*/ERF*s in the durum wheat (*Triticum turgidum* ssp. *durum*) genome, which resulted in the identification of 271 genes distributed on chromosomes 1A-7B. By carrying 27 genes, chromosome 6A had the highest number of *TtAP2*s*/ERF*s. Furthermore, a duplication assay of *TtAP2*s*/ERF*s demonstrated that 70 duplicated gene pairs had undergone purifying selection. According to RNA-seq analysis, the highest expression levels in all tissues and in response to stimuli were associated with *DRF* and *ERF* subfamily genes. In addition, the results revealed that *TtAP2/ERF* genes have tissue-specific expression patterns, and most *TtAP2/ERF* genes were significantly induced in the root tissue. Additionally, 13 *TtAP2/ERF* genes (six *ERF*s, three *DREB*s, two *DRF*s, one *AP2*, and one *RAV*) were selected for further analysis via qRT-PCR of their potential in coping with drought and salinity stresses. The *TtAP2/ERF* genes belonging to the DREB subfamily were markedly induced under both drought-stress and salinity-stress conditions. Furthermore, docking simulations revealed several residues in the pocket sites of the proteins associated with the stress response, which may be useful in future site-directed mutagenesis studies to increase the stress tolerance of durum wheat. This study could provide valuable insights for further evolutionary and functional assays of this important gene family in durum wheat.

## 1. Introduction

Various abiotic and biotic stresses, such as plant pathogens, drought, cold, heat, and salinity, have destructive effects on the growth and development of plant species. Plants employ complex regulatory pathways to address various stimuli, which include stress sensing, signal transduction, and regulation of stress-responsive genes and/or proteins eventually manifested at the cellular and physiological levels [1,2]. The modulation of gene expression in response to stresses is highly controlled by specific transcription factors (TFs), which simultaneously mediate the transcription of a vast number of downstream stress-responsive genes [3,4]. TFs have been extensively proposed to be significant regulatory proteins involved in modifying plant growth and the stress response [5,6]. Among the members of multiple TF groups, the members of the APETALA2/ethylene-responsive factor (AP2/ERF) superfamily have multiple functions in plant development and play roles in the biosynthesis of many key metabolites, providing the ability to address environmental stress [7]. Accordingly, understanding the molecular functions of these gene family members can be helpful for the improvement of crop plant compatibility and yield under various unfavorable situations.

AP2/ERF family-related proteins, which compose a large family, play essential roles in plant growth, enlargement, and coping with different stresses such as extreme chilling/heat, drought, and high-salinity conditions as well as infection by viral and microbial pathogens [8,9,10]. A large number of *AP2/ERF* genes have been recognized in various plant species, such as *Arabidopsis* [11], bread wheat [12], soybean [13], rice [9], *Brassica napus* [14], *Medicago sativa* [15], pineapple [16], *Camptotheca acuminata* [17], orchardgrass [18], foxtail millet [19], sorghum [20], ginseng [21], and *Gynostemma pentaphyllum* [22]. Three main clades of AP2/ERF family members have been determined based on the domain number: the ERF subfamily, whose members contain a single AP2 domain; the AP2 subfamily, whose members contain two AP2 domains connected by a 25 amino acid linker; and the RAV subfamily, whose members have a single AP2 domain and an additional B3 DNA-binding motif [19]. AP2 subfamily genes have been reported to be the main regulatory factors involved in organ development, including spikelet meristem differentiation, leaf epidermal cell designation, floral organ patterning, and seed yield [23,24,25]. Members of the RAV group of the AP2/ERF family also have important functions in plant hormone signal transduction and the regulation of responses to biotic and abiotic stimuli [26,27]. Dehydration-responsive element-binding (DREB) members, as important plant-specific transcription factor, also belong to the AP2/ERF superfamily and are involved in response to abiotic stresses [28]. Furthermore, various studies have reported the involvement of DREBs and other ERF members in dealing with water deficit [29,30], cold, and oxidative [31] and salinity [32,33] stresses. Cui et al. reported that 11 *BdAP2/ERF* genes are involved in various developmental and physiological processes during the *Brachypodium distachyon* life cycle [34]. Furthermore, Kavas et al. indicated higher expression levels of *PvAP2-ERF111*, *PvAP2-ERF119*, *PvAP2-ERF72*, and *PvAP2-ERF150* genes in common bean leaf tissues after salt treatment, suggesting the involvement of these genes in salt stress signal transduction [35]. Ma et al. identified a novel AP2/ERF gene from rice, *OsRPH1*, that negatively controls plant height [36]. Additionally, a novel ERF gene, *ZmERF105*, was isolated from maize that positively controls resistance to *Exserohilum turcicum* [37]. In addition, the members of the *AP2/ERF* gene family regulate the biosynthesis of many key metabolites, such as terpenoid indole alkaloids, nicotine, steroidal glycoalkaloids, tanshinone, and phenolic acids [38,39].

The genome of tetraploid wheat (*Triticum turgidum* ssp. *durum*) is approximately 10.45 Gb, and this subspecies is frequently used to provide nutrition via noodles and pasta making. Very few *AP2/ERF* genes have been distinguished and characterized in the durum wheat genome to date [29,30,32,40], and no computational biological evaluation of the AP2/ERF family has been performed in this important crop species. In the present study, members of the *AP2/ERF* gene family were identified in the durum wheat genome, and multiple structural and functional characterizations were carried out. The results of this study provide further insights concerning the regulatory roles of these genes in the development and stress response of durum wheat and provide knowledge on the evolutionary systems and molecular pathways affected by AP2/*ERF*s in durum wheat and its relatives.

## 2. Materials and Methods

### 2.1. Identification of TtAP2/ERF Genes in the Genome of Durum Wheat

For the identification of the members of the *AP2/ERF* gene family, the reference genome of *Triticum turgidum* was obtained from BioProject No. PRJEB22687 from the Ensembl database [41]. A hidden Markov model (HMM) search was conducted via the HMMER 3.0 program with the AP2 domain (PF00847) sequence used as a query (E-value < ×10^−5^), after which the retrieved protein sequences were searched against the content of the Pfam database [42] for the presence of the specific AP2 domain. Additionally, the identified TtAP2/ERF sequences were assessed through the SMART database [43], and any partial or low-quality AP2 domain-containing sequences were identified and removed from further characterization. The corresponding cDNA and genomic sequences of the identified TtAP2/ERF proteins were also distinguished among the sequenced contigs in the durum wheat genome, and the chromosomal location of the *TtAP2/ERF* genes was determined through gene ID searches in Ensembl (PRJEB22687). The ProtParam program of the ExPASy server [44] was utilized to determine the important physicochemical characteristics, such as the molecular weight (MW) and theoretical isoelectric point (*pI*) of the TtAP2/ERF proteins. The CELLO program [45] was also employed to determine the subcellular location of each TtAP2/ERF protein as well as for gene ontology (GO) annotations of the *TtAP2/ERF* genes through the CELLO2GO tool.

### 2.2. Chromosomal Mapping, Gene Duplications, and Estimation of Ka/Ks Values of the Duplicated Pairs

The identified *TtAP2/ERF* genes were mapped onto the durum wheat chromosomes based on their predicted positions in Ensembl by using MapChart software (v. 2.32) [46]. The duplication events across *TtAP2/ERF* genes were detected by alignment of the *TtAP2/ERF* coding DNA sequences through the ClustalX program (v. 2.0) [47], after which BioEdit software (v. 7.2.5) was used to predict the identity matrix between the aligned CDSs [48]. The duplicated gene pairs were ultimately distinguished as genes sharing greater than 85% identity, as a threshold line, at the nucleotide sequence level, and were manually highlighted on the chromosomal map. To estimate the divergence of homologous *TtAP2*s*/ERF*s and the selective pressure against the duplicated genes, DnaSP software (v. 6.0) was applied to calculate the Ks (synonymous) and Ka (nonsynonymous) replacement rate per site between the members of each gene pair [49].

### 2.3. Phylogenetic Analysis and Motif Recognition

To investigate the phylogenetic relationships, the AP2/ERF protein sequences from durum wheat as well as *Oryza sativa* and *Arabidopsis thaliana*, which were retrieved from the Plant TF Database [50], were employed to construct phylogenetic trees based on the neighbor-joining (NJ) method via MEGA software (v. 6.0) [51], and 1000 replicates were also selected for bootstrap analysis to represent the evolutionary history. The TtAP2/ERF protein sequences were also subjected to MEME software (v. 5.2.0) [52] for identification of conserved motifs. Protein sequences of the AP2/ERF family were screened to identify the repression motifs including L/F DLNL/F [53], R/KLFGV [54], and EDLL motif [55] using Clustal Omega [56].

### 2.4. Three-Dimensional Protein Modeling and Molecular Docking via Protein Pocket Sites

Three-dimensional structures related to four candidate TtAP2/ERF proteins from each of the recognized subfamilies including TtAP2/ERF-001 from DREB, TtAP2/ERF-036 from RAV, TtAP2/ERF-220 from AP2, and TtAP2/ERF-251 from ERF were created using iterative template-based fragment assembly simulations in the I-TASSER program [57]. The best models from I-TASSER were further improved by the 3Drefine program [58]. Afterward, the predicted structures were confirmed using a Ramachandran plot by measuring the backbone dihedral phi (ϕ) and psi (Ψ) angles by the RAMPAGE program [59]. To predict the protein pockets and cavities, the refined structures of *TtAP2*s*/ERF*s were subjected to the P2Rank program of PrankWeb software [60] and the CavityPlus tool [61], after which the structures were ultimately visualized via PyMOL (v. 2.4.1) [62].

### 2.5. In Silico Expression Analysis of TtAP2/ERF Genes through RNA-seq Data

To evaluate the expression patterns of the *TtAP2/ERF* genes in different tissues (stem, leaf, grain, root, and spike) of durum wheat and in response to stress, 1 and 6 h after heat stress (40 °C) and drought stress (20% PEG6000), the available RNA-seq data of *TtAP2*s*/ERF*s were retrieved from project PRJEB22687 (SRA accession: SRP149116) from the Ensembl database. The fragments per kilobase of transcripts per million mapped reads (FPKM) expression values in five tissues as well as in response to stimuli (heat and drought) were obtained for the cultivar Svevo and then not expressed genes in all tissues and response to heat and drought stress were eliminated. Finally, log2 transformed to generate heatmaps using TBtools software (v. 1.0) [63].

### 2.6. Plant Materials and Stress Treatments

*Triticum turgidum* (durum wheat) seeds prepared from the Pasteur Biotechnology Institute of Tehran were disinfected by using NaOCl (0.5%) for 15 min and then put into glass dishes with Whatman filter paper, after which the dishes were incubated under a temperature of 25 ± 2 °C, a 16 h photoperiod (5000 lux), and 60 ± 5% relative humidity. After seed germination, four-day-old seedlings were transplanted into aerated hydroponic culture tanks holding 30 L of a sterile half-strength Hoagland nutrient solution (pH 6.0). Sixty seedlings were vertically fixed in each tank. After two weeks, the seedlings were exposed to drought and salinity stresses. Drought and salt stresses were imposed by the addition of PEG 6000 (polyethylene glycol, 15% *w/v*) and NaCl (200 mM) to the nutrient solution, respectively. All treatments involved three biological replicates. Leaf samples were harvested after 6 and 24 h of stress treatments, and unstressed seedlings were used as control samples. Afterward, the collected plant materials were directly frozen in liquid nitrogen and stored at −80 °C until extraction of their total RNA.

### 2.7. RNA Extraction and RT-qPCR-Based Expression Assays of TtAP2s/ERFs

Four wheat seedlings were pooled for RNA isolation. For extraction of the total RNA from the samples, TRIzol reagent (Invitrogen, Carlsbad, CA, USA) was used according to the manufacturer’s recommendations. To eliminate genomic DNA, the extracted RNA was treated with *DNase I* (Thermo Fisher Scientific, Wilmington, MA, USA), after which the quality and quantity of the RNA samples were checked via an Implen N50 NanoPhotometer (Implen, Munich, Germany). Complementary DNA (cDNA) synthesized using an M-MULV reverse transcriptase kit (Thermo Fisher Scientific, Wilmington, MA, USA) according to the manufacturer’s recommendations.

From the results achieved with the RNA-seq data re-analyzed in the previous section, 13 genes including six *ERF*s (*TtAP2/ERF-009*, *TtAP2/ERF-025*, *TtAP2/ERF-070*, *TtAP2/ERF-104*, *TtAP2/ERF-107*, and *TtAP2/ERF-214*), three *DREB*s (*TtAP2/ERF-176*, *TtAP2/ERF-206*, and *TtAP2/ERF-227*), two *DRF*s (*TtAP2/ERF-185* and *TtAP2/ERF-232*), one *AP2* (*TtAP2/ERF-271*), and one *RAV* (*TtAP2/ERF-099*) have been selected because of their potential involvement in drought and salinity stress responses, and their expression levels were further investigated in a qPCR analysis. The specific primers for the 13 candidate *TtAP2*s*/ERF*s and wheat *Actin* gene (AB181991.1), which served as a housekeeping gene for normalization of the RT-qPCR data, were designed using Primer3 online software [64] (Table S1). RT-qPCR was performed in 20 µL reactions containing Maxima SYBR Green/ROX qPCR Master Mix (Thermo Scientific, Wilmington, MA, USA) according to the manufacturer’s recommendations through a CFX96 RT-qPCR detection system (Bio-Rad, Hercules, CA, USA). Two-step thermal cycling was used for RT-qPCR, according to the company’s instructions (initial activation step of 2 min at 50 °C and 10 min at 95 °C, followed by 40 cycles of 95 °C for 15 sec and 60 °C for 1 min). The relative expression levels of the 13 candidate *TtAP2/ERF* genes were calculated using the 2^−∆∆CT^ method [65].

## 3. Results

### 3.1. Identification of TtAP2/ERF Genes and Their Chromosomal Positions in the Durum Wheat Genome

**Figure 1:**
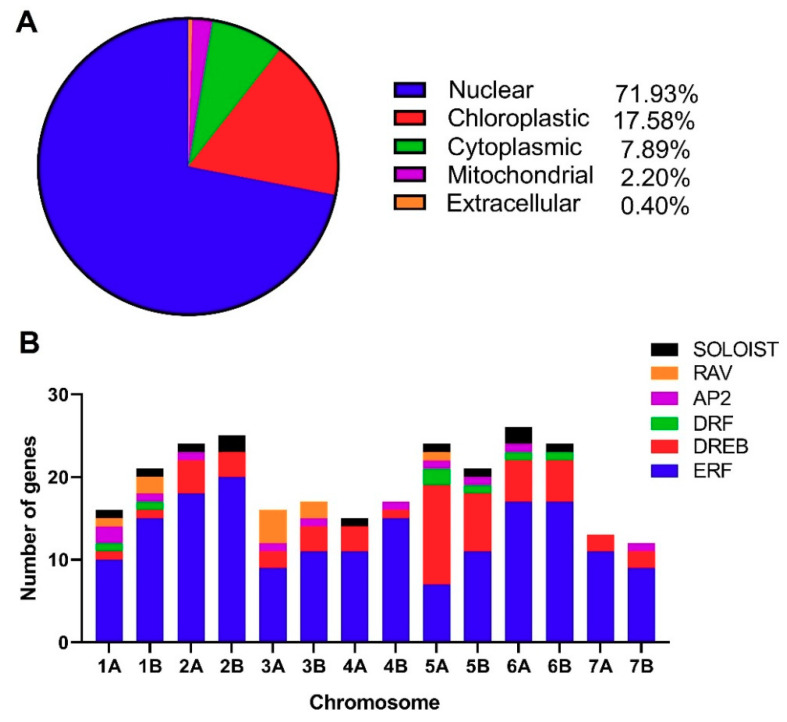
Subcellular localization of TtAP2/ERF proteins in durum wheat (**A**) and distribution of the *TtAP2/ERF* genes on the durum wheat chromosomes in both the A and B subgenomes (**B**).

**Figure 2:**
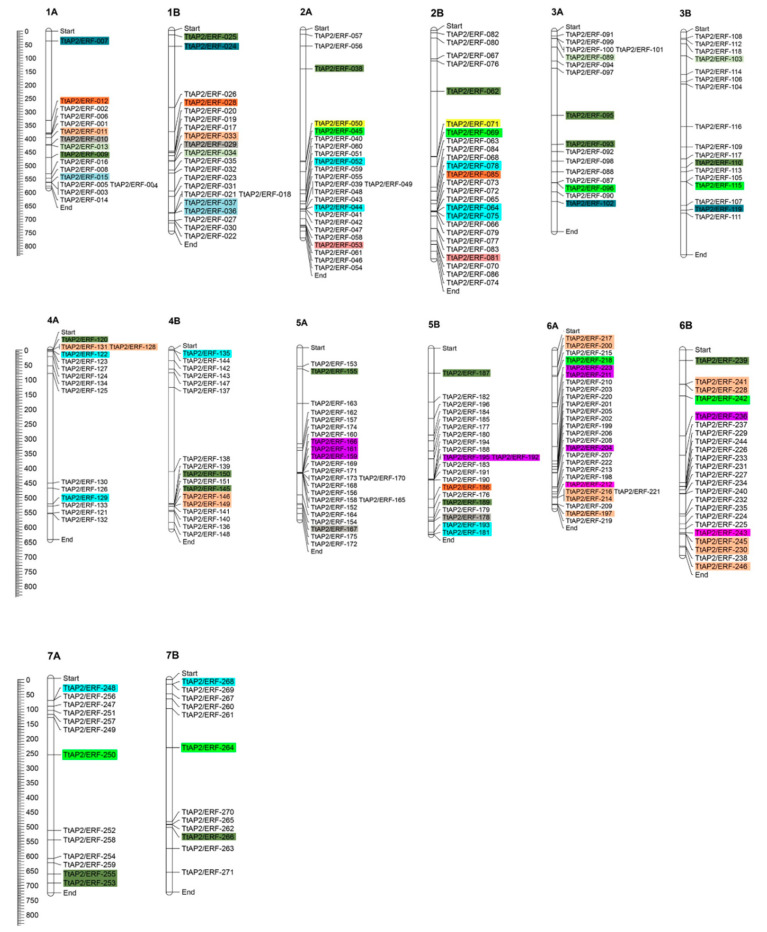
Chromosomal distribution of 271 non-redundant putative *TtAP2/ERF* genes predicted from the *T. turgidum* genome. The predicted duplicated pairs are highlighted in the same color.

The deduced protein sequences of *T. turgidum* investigated via HMMER software led to the identification of 271 non-redundant putative TtAP2/ERF proteins (Table S2). The recognized genes were named according to their chromosomal location order in the A and/or B subgenomes of durum wheat as *TtAP2/ERF-001* to *TtAP2/ERF-271*. There were 237 *ERF* (including 36 DREBs, 16 DREB-related factors (so-called *DRF*s), and 185 ERFs), 11 AP2, 10 RAV, and 13 SOLOIST (AP2/ERF-like) subfamily-related proteins in the recognized *TtAP2/ERF* gene family (Table S2). The putative proteins of *TtAP2*s*/ERF*s ranged from 80 (TtAP2/ERF-116) to 702 (*TtAP2/ERF-102*) amino acids in length, with molecular weights (MWs) ranging from 9.241 (*TtAP2/ERF-116*) to 153.177 kDa (*TtAP2/ERF-103*) and theoretical isoelectric points (pI) ranging from 4.34 (*TtAP2/ERF-149*) to 12.03 (*TtAP2/ERF-014*), revealing an acidic nature of most of the putative TtAP2/ERF proteins (~65.68%). Subcellular localization analysis based on CELLO results indicated that the majority of *TtAP2*s*/ERF*s (~72%) were localized in the nucleus, while 17.6% of TtAP2/ERF proteins were predicted to be localized in the chloroplast. Furthermore, 7.9, 2.2, and 0.4% of the proteins were also detected to be cytoplasmic, mitochondrial, and extracellular *TtAP2*s*/ERF*s, respectively (Figure 1A). Among the predicted *TtAP2*s*/ERF*s, 136 genes were mapped onto the durum wheat B subgenome, and 135 genes were predicted to be localized onto the A subgenome (Figure 2). By carrying 27 genes, chromosome 6A had the highest number of *TtAP2*s*/ERF*s, while chromosomes 2B, 2A, 5A, and 6B contained 25, 24, 24, and 23 genes, respectively. Chromosomes 1B and 5B also revealed substantial numbers of *TtAP2*s*/ERF*s, each carrying 21 genes, and chromosomes 7A and 7B had the minimum number of *TtAP2*s*/ERF*s (Figure 1B and Figure 2).

### 3.2. Conserved Amino Acid Residues in the DNA-Binding Domains of TtAP2/ERF Proteins

**Figure 3:**
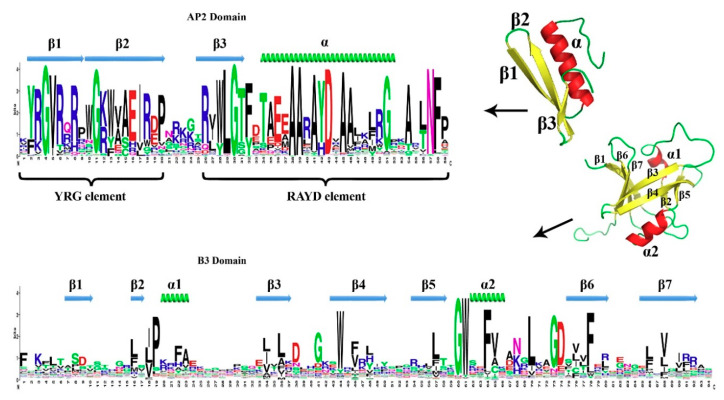
Molecular structure of the specific AP2 and B3 domains found in the 271 non-redundant putative TtAP2/ERF proteins predicted from the *T. turgidum* genome. The abundance of each amino acid is represented by a sequence logo and the secondary and the tertiary structure of each of the domains has been predicted using the PDB database and the I-TASSER program.

To analyze the conservation of the TtAP2/ERF proteins from different subfamilies, their specific AP2 (SM000380) and B3 (SM01019) domains were aligned to generate specific sequence logos (Figure 3). AP2 is characterized by two conserved regions called YRG and RAYD. As regards the AP2 domain, the structure prediction revealed three β-sheets (β1, β2, and β3) and one α-helix region that shared significant amino acid similarity within two YRG and RAYD elements (Figure 3). In particular, the amino acid residues 4G, 11G, 29L, 30G, 38A, 39A, 43D, and N57 were highly conserved among *TtAP2*s*/ERF*s. The structure of β-sheet 1 contained a conserved GVR element along with several sequences. Moreover, β-sheet 2 revealed a conserved EIR element with several substitutions, including 16H for 16E, 17V for 17I, and 18W for 18R. Our results suggested that the amino acid residues of EVR were present only in the *ERF* and *DRE*B genes. In addition, β-sheet 3 revealed a conserved WLG element, which was highly conserved between *T. durum* and other species, such as *Arabidopsis*. Furthermore, the α-helix structure contained a consensus sequence (positions 38–43) AAxA[YH]D, which is highly conserved among the RAYD elements (Figure 3). The conserved Ala45, an important residue for binding to GCC-box and DRE *cis*-elements [14,66], was also fully conserved among all members of *TtAP2*s*/ERF*s. Moving to the B3 domain, characterized by seven β-sheets and two α-helices (Figure 3), the amino acid residues 19P, 36L, 38D, 41G, 44W, 60G, 61W, 64F, 65V, 70L, 73G, 74D, and 78F were highly conserved among all TtAP2s/RAVs (Figure 3). The N-terminal region of TtAP2/RAV proteins contained an AP2 DNA-binding domain, while a highly conserved B3 domain existed in the C-terminal region. The B3 domain comprised seven β-sheets and two α-helices (Figure 3).

### 3.3. Phylogenetic Relationships and Conserved Motifs

Phylogenetic analyses are necessary to understand the evolutionary history of plant lineages. Phylogenetic analysis revealed the clustering of all *TtAP2*s*/ERF*s into 19 different groups; clades I to X and XIV to XVI contained multiple kinds of *ERF*s, clades XI and XII comprised *DREB*s, clade XIII contained DREB-related factors (*DRF*s), clade XVII comprised RAV proteins, clade XVIII contained AP2s, and, lastly, clade XIX contained SOLOIST members (Figure S1). Based on the conserved protein motifs, 15 motifs (Figure S1) were identified of which five of them (motifs 1, 2, 3, 4, and 10) represented the AP2 domain in the TtAP2/ERF structure (Figure S1 and Table S3). Motif 2 and motif 1 contained the important parts of the AP2 domain, the YRG and RAYD elements, respectively, and motifs 4 and 3 were related to the N-terminal region of YRG and the C-terminal region of RAYD elements, respectively. The motif combination comprising motif 4, motif 2, motif 1, and motif 3 was related to the AP2 domain catalytic activity and was predicted to exist in approximately all *TtAP2*s*/ERF*s, except for the XVII, XVIII, and XIX clade-related proteins that contained two or three AP2 catalytic motifs. Motif 8 and motif 14 were present in the B3 domain in all RAV-related proteins (Figure S1). The AP2/ERF proteins belonging to the same groups also had several conserved motifs beyond the AP2 domain region. For instance, motifs 11, 14, and 8 were shared by all members in the RAV subfamily, and motifs 4, 9, and 11 were also present in AP2 subfamily-related proteins (Figure S1). The predicted SOLOIST proteins had a modified combination of the AP2 protein motifs that were in accordance with the reason underlying their nomenclature. Additionally, motif 5 and motif 6 were predicted only in some members of ERF proteins in clade II, and motif 12 and motif 13 existed only in *ERF*s from clades V and XV, respectively. Hence, the motif structures are somewhat conserved in each AP2/ERF clade, which is in line with the conserved and specific functions of the proteins in these clusters.

**Figure 4:**
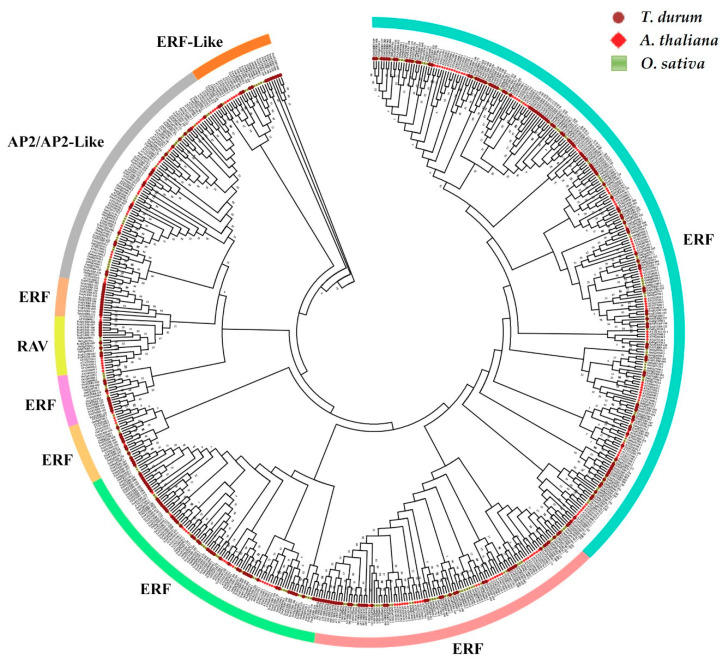
Phylogenetic analysis of AP2/ERF proteins from durum wheat, rice, and Arabidopsis using MEGA 6 software based on the NJ method.

The evolutionary relationships of the *TtAP2/ERF* genes were further assayed by phylogenetic analysis of all the *TtAP2/ERF* members and the rice and *Arabidopsis AP2/ERF* gene family members. According to the results, the *AP2/ERF* genes could be clustered into nine major groups, six ERF clades, a RAV clade, an AP2/AP2-Like clade, and an ERF-Like clade, based on their domain structure as described above and on their evolutionary history (Figure 4). With respect to the classification criteria in *Arabidopsis* and rice [67], the *ERF*s could be further divided into DREB and ERF subfamilies. The AP2 group could also be divided into two AP2-like (SOLOIST) and AP2 subclades. In addition, the bootstrapping magnitudes of the nodes of this phylogenetic tree were substantial in the ERF clades, suggesting that the cellular function of these proteins from various plant species was relatively evolutionarily conserved.

### 3.4. Gene Ontology Annotations

**Figure 5:**
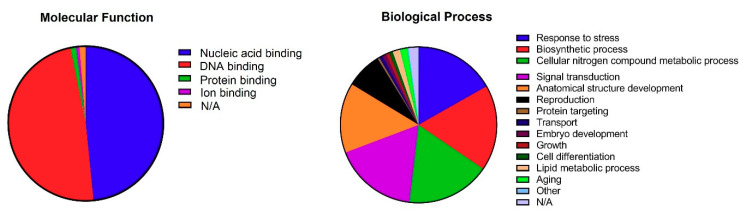
Molecular functions and biological processes of members of the *TtAP2/ERF* family based on gene ontology (GO) analysis.

Evaluation of the biological processes mediated by *TtAP2*s*/ERF*s indicated that the same percentage of proteins were involved in cellular nitrogen compound metabolic processes (~19%) and biosynthetic processes (~19%). Among the TtAP2/ERF family proteins, ~18 and 17.6% of members showed potential involvement in signal transduction and response to stimuli, respectively, during the durum wheat life cycle (Figure 5). Furthermore, the TtAP2/ERF proteins were found to be involved in the regulation of multiple aspects of developmental processes, such as anatomical structure development (~15%), regulation of growth (~4%), embryo development (~2%), and cell differentiation (~1%). Moreover, the involvement of *TtAP2*s*/ERF*s in reproduction (~8%), lipid metabolic processes (~2%), and aging control (~2%) was predicted through gene ontology enrichment analysis. In the context of molecular functions, as expected the 96% of the TtAP2/ERFs showed GO IDs related to nucleic-acid binding activity (Figure 5). The molecular processes regulated by TtAP2/ERF proteins significantly demonstrated that most of these proteins exert sequence-specific DNA-binding transcription factor activity. The potential involvement of several TtAP2/ERF proteins in protein binding (2%) and ion binding (2%) were also predicted to be molecular functions in the cell.

### 3.5. Identification of Duplicated Gene Pairs with Estimation of Ka/Ks Ratios

Seventy duplicated gene pairs clustering into the 13 groups were identified in the *TtAP2/ERF* family based of phylogenetic results, and the same duplicated regions between paralogous pairs were found (Figure 2). Among the duplicated clades, clades 10, 1, and 3 revealed substantial numbers of duplication events, with 29, 11, and 8 gene pairs, respectively (Table S4). The highest number of duplicated gene pairs was observed on chromosomes 6A and 6B, with nine duplicated gene pairs. In this study, the *Ka*/*Ks* values varied from 0.0035 to 0.9936. Several of the duplicated gene pair blocks, such as *TtAP2/ERF-036–TtAP2/ERF-037* and *TtAP2/ERF-064–TtAP2/ERF-075*, were collinear based on analysis of intra-species synteny. All the duplicated gene groups in the *TtAP2/ERF* gene family of durum wheat have been influenced by intense purifying selection because of the estimated *Ka/Ks* values <1. Based on phylogenetic analysis, annotations, and motif structures, it was revealed that the genes in a duplicated pair can be functionally conserved. For instance, the duplicated pairs *TtAP2/ERF-010-TtAP2/ERF-029* and *TtAP2/ERF-167-TtAP2/ERF-178* were related to the phylogenetic clade XIX and the SOLOIST proteins, which showed similar motif patterns (Figure S1 and Table S4).

### 3.6. Homology Modeling of TtAP2/ERF Proteins and Docking Assays of Their Pocket Sites

**Figure 6:**
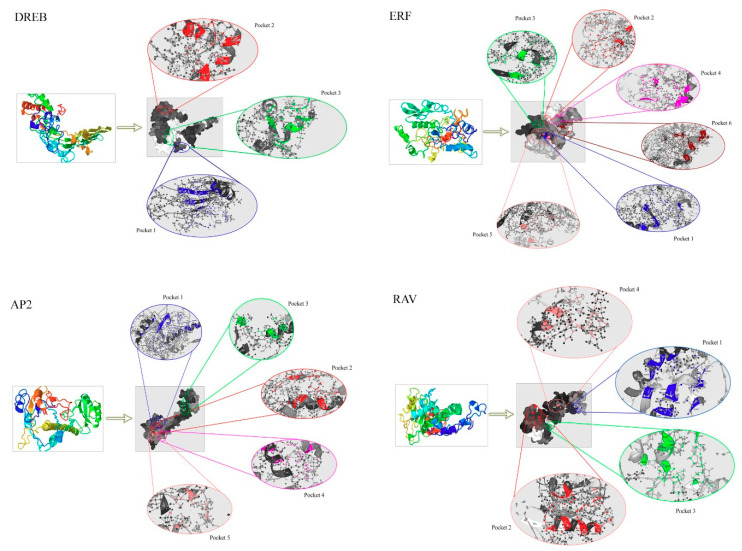
Predicted 3D structure of candidate TtAP2/ERF proteins in durum wheat. Docking analysis of the major protein pocket sites was also performed before each 3D model was constructed.

Three-dimensional models of four candidate TtAP2/ERF proteins (one member from each of the DREB (TtAP2/ERF-001), ERF (TtAP2/ERF-251), RAV (TtAP2/ERF-036), and AP2 (TtAP2/ERF-222) subfamilies) were assembled using the PDB database and the I-TASSER program. The 3D structures showed the presence of the conserved AP2 domain (approximately 60–70 amino acids) within all the *TtAP2*s*/ERF*s; the domain had a typical three-dimensional frame comprising three antiparallel β-sheets followed by a parallel α-helix (Figure 6). The N-terminal region of the TtRAV protein contained one AP2 domain, along with a highly conserved B3 domain that existed in the C-terminal region and that was comprised of seven β-sheets and two α-helixes (Figure 6).

Topographic features of TtAP2/ERF proteins were assessed through the P2Rank program; the major pockets of which are presented here as multiple colored regions (Figure 6). Three, six, four, and five major pockets were predicted as binding regions/active sites in the candidate proteins from the DREB, ERF, RAV, and AP2 clusters, respectively. The amino acid residues present in the pocket sites of the TtAP2/ERF proteins partially differed in each subfamily, although Mg or Ca ions were present in the center of the active sites of all the predicted TtAP2/ERF protein models. SER, GLY, HIS, PRO, GLU, TYR, and ARG amino acids were identified as the important binding residues in the predicted pocket sites of the DREB and ERF proteins (Figure 6); these findings strongly indicate the potential roles of these proteins in the response to stress as well as growth and development modification in durum wheat. Investigations of the predicted pocket sites of RAV and AP2 family-related proteins also demonstrated some parts rich in the stress-responsive residues GLY, PRO, HIS, and SER as well as growth-regulating amino acids LEU, VAL, GLU, ALA, and TYR (Figure 6). Based on our results, the important amino acids found in the pocket sites of all the candidate TtAP2/ERF proteins may indicate the importance of these residues in the positioning onto the DNA molecule and, ultimately, the cellular function associated with various developmental and defensive processes.

### 3.7. Assay of TtAP2s/ERFs Expression in Multiple Tissues and under Abiotic Stimuli via RNA-Seq

**Figure 7:**
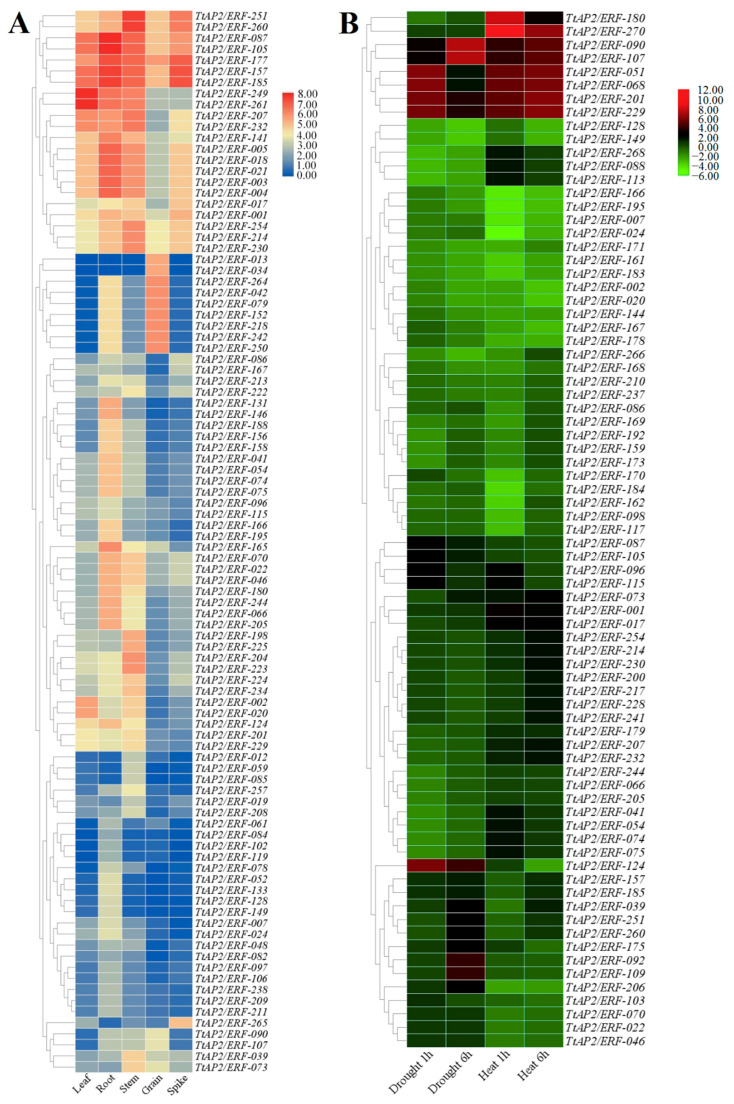
Expression heatmaps of putative *TtAP2/ERF* genes in five different tissues (namely leaf, root, stem, grain, and spike) of durum wheat (**A**) and under drought (1 and 6 h) and heat stresses (1 and 6 h) conditions (**B**).

The expression levels of the *TtAP2/ERF* genes were evaluated under normal growth conditions in multiple tissues as well as during stress conditions via available RNA-seq datasets. The results revealed that *TtAP2/ERF* genes have tissue-specific expression patterns (Figure 7A). According to the transcript levels, *TtAP2*s*/ERF*s could be divided into different expression groups that contained genes preferentially expressed in all or one of the following tissues: grain, root, leaf, spike, and stem tissues (Figure 7A). The expression levels related to the seven *TtAP2/ERF* genes were significantly high in all five tissues, including the expression of five and two genes from the *ERF* and *DRF* subfamilies, respectively, suggesting control of a broad set of genes at the transcriptional level. According to the results, it was also found that *AP2/ERF* genes are less expressed in spike than other tissues (Figure 7A). Seven *ERF* genes (*TtAP2/ERF-042, TtAP2/ERF-079, TtAP2/ERF-152, TtAP2/ERF-218, TtAP2/ERF-242, TtAP2/ERF-250*, and *TtAP2/ERF-264*), and two *DREB* genes (*TtAP2/ERF-013* and *TtAP2/ERF-034*) were specifically expressed in durum wheat grain tissue. The largest tissue-specific expression group included genes that were significantly expressed in the root and stem tissues (Figure 7A).

RNA-seq data were also employed to further verify the expression of the identified *TtAP2/ERF* genes after 1 h and 6 h of heat and drought stresses in leaf tissues of durum wheat. A total of six *TtAP2/ERF* genes from the ERF subfamily (*TtAP2/ERF-090*, *TtAP2/ERF-107*, *TtAP2/ERF-051*, *TtAP2/ERF-068*, *TtAP2/ERF-201*, and *TtAP2/ERF-229*) were significantly upregulated under all stimuli (Figure 7B). Based on the results, most genes were downregulated in response to drought and heat stresses. The mRNA levels of three genes, such as *TtAP2/ERF-124* belonging to the DREB subfamily, *TtAP2/ERF-124* and *TtAP2/ERF-092* from the *ERF* subfamily increased only in response to drought conditions and could be considered drought-responsible *TtAP2/ERF* genes. Furthermore, the transcript levels of two *TtAP2/ERF* genes, including *TtAP2/ERF-180* and *TtAP2/ERF-270* from the *ERF* gene subfamily, were significantly upregulated only after heat stress exposure, suggesting their involvement in the heat response. However, some *DREB*s were downregulated in response to stress, suggesting an acclimation response involving members of this subfamily, which may assist in normalizing cell osmotic potential and, eventually, durum wheat resistance to stimuli. It should be mentioned that the response to stress by the ERF protein-encoding genes was considerable in comparison with that of the other related subfamily genes. Furthermore, the transcript levels of the genes encoding DREB proteins and ERF proteins were high in response to stimuli.

### 3.8. Identification Repression Motifs in TtAP2/ERFs

**Figure 8:**
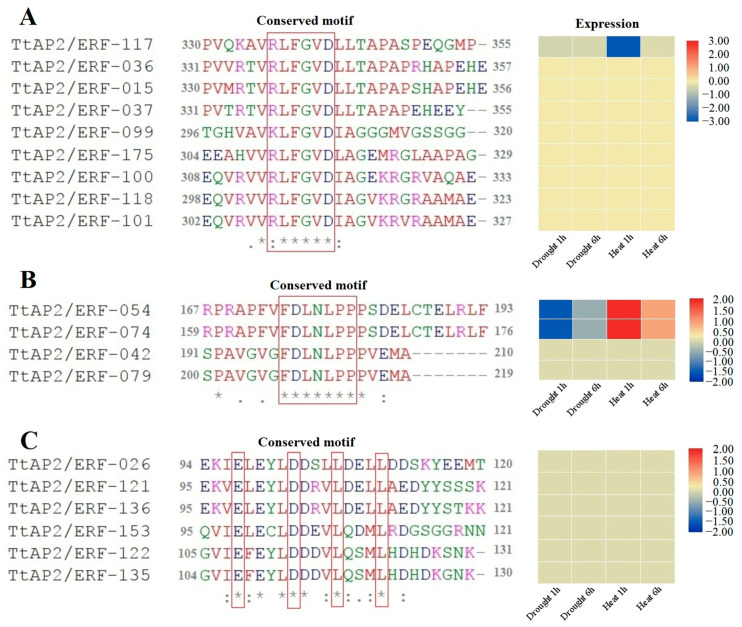
List of TtAP2/ERF genes contained repressor motifs with their expression. List of genes containing [R/K]LFGV motif (**A**), FDLNLPP motif (**B**), and EDLL motif (**C**).

Protein sequences of *TtAP2/ERF* genes were screened to identify the repression motifs, [R/K]LFGV, FDLNLPP, and EDLL. The results showed that nine genes belonging to the RAV subfamily contain the [R/K]LFGV motif, most of which (except for *TtAP2/ERF-117*) were not induced in response to drought and heat stress (Figure 8A). Additionally, four proteins belonging to ERF subfamily had a FDLNLPP motif, and two genes of them, *TtAP2/ERF-054* and *TtAP2/ERF-074*, were involved in early response to drought and heat stress (Figure 8B). Besides, motif EDLL was identified in six ERF proteins that were not expressed under drought and heat stress (Figure 8C).

### 3.9. Expression Levels of TtAP2s/ERFs after Stimulus Exposure According to RT-qPCR

**Figure 9:**
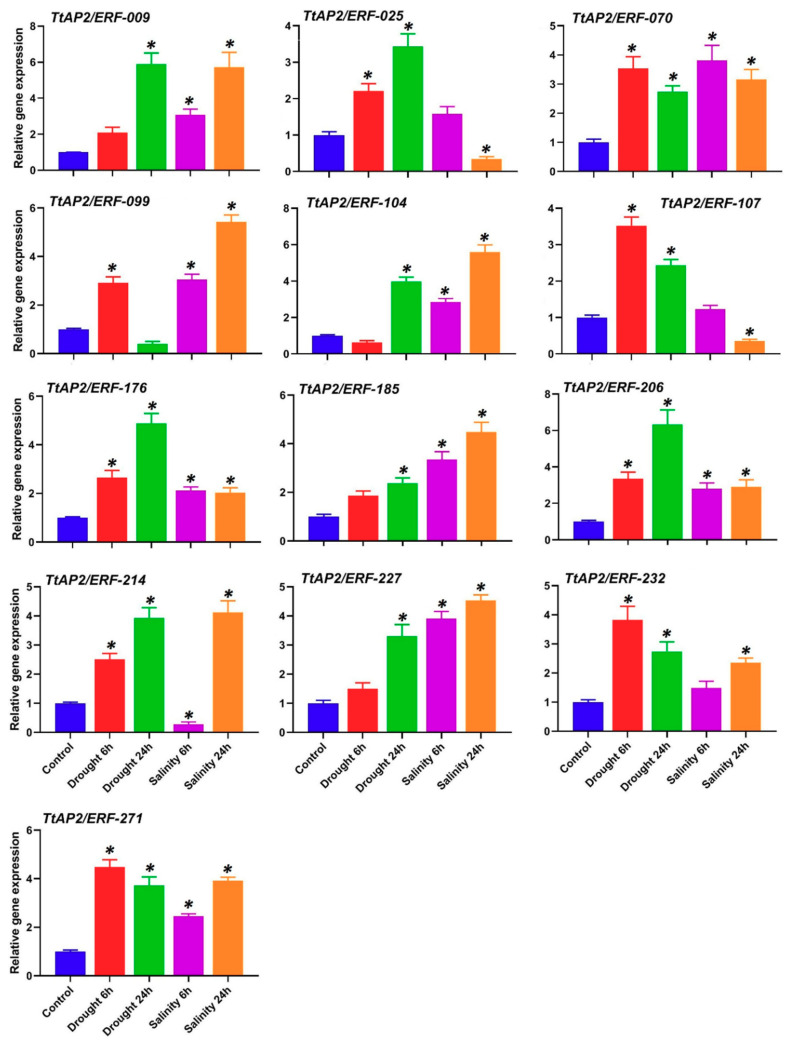
Relative expression levels of 13 candidate *TtAP2/ERF* genes after drought and NaCl stress. The values are given as the means ± SDs of three biological replicates, and the * above the bars show a significant difference between the applied treatments and the control at *p* < 0.05 (according to Student’s *t*-test).

From the results achieved with the RNA-seq data re-analyzed in the previous section, 13 genes have been selected because of their potential involvement in drought and salinity stresses responses, and their expression levels were further investigated in a qPCR analysis. Thirteen *TtAP2*s*/ERF*s (including six *ERF*s, three *DREB*s, two *DRF*s, one *AP2*, and one *RAV*) were selected for further assays of their potential in coping with drought and salinity stresses in durum wheat leaf tissue. The *TtAP2/ERF* genes belonging to the *DREB* gene subfamily (including *TtAP2/ERF-176*, *TtAP2/ERF-206*, and *TtAP2/ERF-227*) were markedly induced under both drought-stress and salinity-stress conditions compared with those in the control sample (Figure 9). The expression levels of the candidate *DRF* genes (including *TtAP2/ERF-185* and *TtAP2/ERF-232*) also increased in response to each stimulus and confirmed the RNA-seq results; *TtAP2/ERF-185* was significantly upregulated in response to salinity-stress conditions, but the transcript levels of *TtAP2/ERF-232* increased under drought stress exposure compared with the control conditions. The transcript levels of a candidate *RAV* gene, *TtAP2/ERF-099*, tended to increase in response to salinity stress over time, but after an approximately three-fold increase after the initial drought stress imposition, the levels significantly decreased in response to the long-term stress compared to those under the control conditions (Figure 9). In contrast to the RNA-seq results, the expression assay of the *RAV* gene *TtAP2/ERF-*099 and *AP2* clade-related gene *TtAP2/ERF-271* revealed a considerable increase in expression in response to each stimulus. Some candidate *TtAP2*s*/ERF*s belonging to the ERF group, such as *TtAP2/ERF-025* and *TtAP2/ERF-107*, were upregulated with increasing drought stress, while significant decreases were found after long-term salinity exposure in comparison with the results of the control sample. The transcript levels of two *ERF* genes, *TtAP2/ERF-009* and *TtAP2/ERF-070*, substantially increased over time in response to all stimuli, suggesting that they have critical roles in stress adaptation (Figure 9). The mRNA levels of *TtAP2/ERF-104* and *TtAP2/ERF-214* significantly decreased during the early drought and salinity stress treatments, respectively; however, their expression levels increased after 24 h of drought and salinity stress. Overall, our findings revealed that most of the selected *AP2/ERF* genes are involved in the response to salinity and drought stress, although their expression levels varied according to the type and duration of stress.

## 4. Discussion

Systematic evaluation of the diverse gene/protein families involved in the regulation of multiple developmental processes and stimuli can provide insights into the critical regulatory mechanisms of different plant genomes. A vast number of previously published reports have shown extensive involvement of *AP2/ERF* transcription factors in plant growth and development and critical cellular metabolism processes [15,68,69]. In the present study, 271 *AP2/ERF* genes were identified in the durum wheat genome, the number of which was lower than that reported in *Brassica napus* [14], with 321 members; however, 271 was greater than the 171 *AP2/ERF* genes in *Setaria italica* [19], the 141 genes identified in *B. distachyon* [34], the 122 genes in *Arabidopsis* [67], the 139 genes in rice [67], and the 117 genes in bread wheat [12]. Based on this, it could be hypothesized that the number of genes is directly correlated with the genome size and ploidy level in plants. In addition to ploidy level and genome size, various distributions of the *AP2/ERF* genes in durum wheat compared to the other cereal species may be determined by gene duplication events to establish certain biological roles, such as resistance to stimuli. The extensive variation predicted for *TtAP2/ERF* gene lengths and the physicochemical properties of these proteins may suggest that the durum wheat genome underwent a significant evolutionary change [70]. The majority of *TtAP2*s*/ERF*s are predicted to be nuclear proteins, suggesting their critical roles in transcriptional adjustment and signal transduction [71].

Several credible pairs of homologous AP2/ERF proteins with the same cellular functions were distinguished in this study because of the high bootstrap values predicted among some internal branches of the phylogenetic constructed tree. Identification of cellular functions according to phylogenetic relationships is frequently reported as a rational systematic approach for exploration of orthologous genes in various plant species [19,72,73]. Accordingly, the *TtAP2*s*/ERF*s belonging to each subfamily in durum wheat revealed similar motif structures and regulatory roles compared with those of their orthologs from other plant species, such as *Arabidopsis* [74] and *B. distachyon* [34]. In the current study, some conserved motifs in TtAP2/ERF proteins were predicted to lie in the regions outside the DNA-binding domain; moreover, it should be mentioned that both the factors affecting transcriptional modification and nuclear localization-related domains can be found in these regions [75]. The predicted similarity in motif structures may result from recent segregation events across the durum wheat genome and its evolutionary process [76]. Some close association of *TtAP2/ERF* subfamily members, especially *DRF*, *DREB*, and *RAV* members, with their related counterparts from the *Arabidopsis* and rice genomes may reveal remarkable similarity in terms of expression and regulatory functions [19].

Gene duplication events have substantial roles in the evolutionary expansion of gene families in terms of the formation of novel genes, which supports organisms adapting to diverse conditions [77]. Tandem, whole-genome, or segmental duplications constitute the proposed models of gene duplications in plant species [77]. In our study, we identified 13 groups of duplicated genes in *TtAP2/ERF* genes dispersed in segmentally duplicated blocks, indicating that the expansion of the *TtAP2/ERF* gene family may be the result of a high number of duplication events [77]. Furthermore, many of the duplicated gene pair blocks were collinear, suggesting that these duplication events may be derived from chromosome segmentation events or large-scale duplication/triplication events [78]. Ka/Ks values are known as an index for selection pressure [77]. In the current study, the mean Ka/Ks value of duplicated *TtAP2/ERF* gene pairs was predicted to be less than 1 (0.4773), strongly supporting that the *TtAP2/ERF* gene family survived extreme purifying selection pressure by substitution elimination and high selective pressure by natural selection throughout the evolutionary process [77,79]. Genes with conserved functions and/or pseudogenization may be generated by purifying selection [80].

Accordingly, molecular modeling suggested that all the predicted structures of proteins were highly consistent, which may provide an elementary reason for understanding the molecular functions of TtAP2/ERF proteins. Our findings were consistent with the results of previous studies on the 3D homology of AP2/ERF proteins [81]. The AP2 subfamily members contain two AP2 domains separated by a linker sequence of approximately 25 amino acids, which is responsible for the positioning of the DNA-binding domains [82] and is well illustrated in the TtAP2 protein structure (Figure 6). Previous studies of the AP2 domain demonstrated the presence of two important regions, namely, RAYD and YRG [19], which were also found in durum wheat TtAP2s/ERFs in the present study. The YRG region includes 20 amino acids in the β1 sheet of the AP2 domain, which has been shown to play a critical role in establishing direct contact with the DNA molecule [83]. Conversely, the RAYD region contains approximately 40 amino acids in the α-helix region and is involved in protein–protein interactions (see Figure 3). Moreover, various reports have also indicated that the RAYD region is required in DNA binding for interactions of the hydrophobic surface of the α-helix with the DNA major groove [83].

Protein surface pockets and interior cavities are considered the topological and geometric characteristics of protein structures that are essential for DNA–protein interactions and enzymatic activities [84]. The interaction specificity of proteins is altered through the dynamics of protein-binding pockets, which contribute to the structural flexibility of the binding process [85]. As such, changes in the surface pocket topography of the modeled *TtAP2*s*/ERF*s can contribute to the functional plasticity of *TtAP2*s*/ERF*s. The high amount of the GLY and PRO amino acids in the pocket sites of the modeled proteins suggest that the amino acid composition (along with the genetic aspects) work to reduce the environmental effects on protein structure and function [86]. It has been reported that GLY/PRO-rich proteins have important roles in plants in response to various abiotic and biotic stress situations by altering the expression of other genes [87]. The presence of several important amino acids, such as SER, LEU, VAL, and PRO, in the protein pocket regions can significantly alter various protein functions in response to adverse conditions [88,89]. Additionally, the CYS and LYS residues observed in pocket 4 and pocket 6 of the ERF protein, respectively, may suggest an important role of these proteins in sulfur metabolism and starch biosynthesis [90]. Furthermore, the cavity region in the TtRAV protein also revealed CYS residues, suggesting a potential role of *TtAP2*s*/ERF*s of these clades in the modulation of sulfur metabolism through regulation of the sulfotransferases [91]. Prediction of the TRP and GLU residues in all of the modeled TtAP2/ERF active sites also demonstrates their important potential involvement in immunity during the hypersensitive response to hemibiotrophs [92]. Our results also suggested that the composition of amino acids in the TtAP2/ERF protein domains was extremely conserved and that some amino acid variations may amend the classification of the genes.

In the present study, in silico expression analysis of all 271 *TtAP2/ERF* genes in five major tissues via RNA-seq datasets demonstrated differential expression of these genes. Expression profiling of tissue-specific *TtAP2*s*/ERF*s can facilitate the combined use of these genes in transcriptional regulation in various tissues and/or organs, while ubiquitously transcribed members can alter the transcription of many genes [21,93]. Tissue-specific expression heatmaps can provide important information concerning the overexpression of *TtAP2*s*/ERF*s in multiple tissues to provide information concerning resistance to stimuli in durum wheat. These changes in the magnitude of expression of transcripts encoding *AP2/ERF*s that participate in signaling and protein targeting mechanisms suggest that the pathway of these TFs involves highly coordinated interactions of both development- and defense-related signals [5,7]. Regarding the results, most of the genes with a tendency to be expressed in specific tissues are members of the *ERF* and *DREB* subfamilies, which may reveal the important roles of these genes in the regulation of development and the integration of multiple organs and/or tissues [68,69]. The in silico transcription assay of the *AP2/ERF* gene family from durum wheat can provide new empirical transcriptome references for improving Triticeae agronomic properties [70] and insights into transcriptional programming during multiple situations.

It has been reported that TtAP2/ERF proteins play important roles in mediating signal transduction pathways and responses to various stresses in durum wheat, and this has been reported by several researchers [19,34,94]. Based on our RNA-seq analysis results, a total of 83 *TtAP2*s*/ERF*s were up- or downregulated under stress, indicating that they are largely involved in the response to stimuli. Genes of the *ERF* subfamily were more highly expressed than were *AP2* genes in response to drought and salinity stresses. For instance, six *TtAP2/ERF* genes from the ERF subfamily (*TtAP2/ERF-090, TtAP2/ERF-107, TtAP2/ERF-051, TtAP2/ERF-068, TtAP2/ERF-201*, and *TtAP2/ERF-229*) were highly upregulated under both drought and heat stress conditions. Several key cis-regulatory elements such as the dehydration-responsive element (DRE) with conserved sequence [AG]CCGAC, ABA-responsive element (ABRE B) with conserved sequence TCCACGTCTC, re2f-1 element with conserved sequence GCGGGAAA, and ACGT motif with conserved sequence GTACGTG were observed in the promoter site (−1500 bp from start codon) of upregulated genes under both drought and heat stresses (data not shown). The presence of these common regulatory elements in the promoter region of upregulated genes seems to affect the induction of these genes in both drought and heat stress conditions. In agreement with our findings, Cui et al. also suggested that, compared with those of *AP2s*, the expression levels of *ERF* subfamily members from *B. distachyon* were more induced after heavy metal, cold, drought, and salt exposure [34]. These differences in expression may be due to the longer exons and/or lack of introns in ERF subfamily genes, which may lead to a faster response and greater expression of these genes during the stress response compared to that of the AP2 subfamily genes in durum wheat [81,94]. It has been widely suggested that AP2/ERF proteins can bind to GCC-box or DRE motifs through their MG metal ion in the AP2 domain, altering the target gene expression under stress conditions [19,20,95]. Previous studies have also illustrated that the genes encoding DREB proteins are significantly cold and drought responsive, so overexpression of these genes could improve drought, salt, and cold tolerance in plants [19,81,95]. Furthermore, DRE is a core sequence of genes involved in the response to drought and cold stresses [3,18,96]. A *DREB2* homolog *Wdreb2* gene, which was firstly isolated from bread wheat, is activated by abiotic stresses such as salt, drought, cold, and exogenous ABA application [97]. In the current study, we found two orthologues of *Wdreb2*, *TtAP2/ERF-001*, and *TtAP2/ERF-017*, from genome of durum wheat, which both orthologues genes showed an upregulation in response to heat stress.

*AP2/ERF*s are considered dynamic genes involved in development and stress response processes. Fan et al. stated that most of the tested *ERF* genes in cassava were upregulated in response to osmotic and salt stresses [82]. The *AtERF53* gene in *Arabidopsis* has been reported to be a drought- and heat-induced transcription factor modulating drought and heat-responsive genes through binding to *cis*-elements, dehydration-responsive elements (DREs), and GCC-boxes of target gene promoters [98,99,100]. In addition, silencing of *ERF54L* and *ERF4L* decreased the salt tolerance of cotton seedlings [101]. Overexpression of *ERF1* from pitahaya (*Hylocereus undatus*) in *Arabidopsis* could increase the activity of antioxidant enzymes and enhance salt tolerance [102]. It was also reported that the insect herbivore-responsive AP2/EFR in *Arabidopsis*, ORA47, has an important function in the adjustment of a suite of genes involved in biosynthesis and phytohormone signal transduction during wounding, in response to MeJA and during drought stress [103,104]. Interestingly, *TtAP2/ERF-227*, *TtAP2/ERF-185*, and *TtAP2/ERF-009* showed close phylogenetic relationships with several genes from *Arabidopsis*, especially genes in the ERF group. According to various studies, most of these homologous genes in *Arabidopsis* are involved in modulating freezing, dehydration, and salinity tolerance via activation of an ABA-responsive network and stress-related genes [105,106,107]. Evidence revealed that *TtAP2*s*/ERF*s might be positively involved in the regulation of salt and drought tolerance in durum wheat. The variable gene transcription patterns obtained from the RT-qPCR-based experiment in the current study showed that most *TtAP2*s*/ERF*s, such as *TtAP2/ERF-271*, *TtAP2/ERF-185*, *TtAP2/ERF-099*, *TtAP2/ERF-206*, and *TtAP2/ERF-070*, may function in mediate as an intricate network for multiple stimulus responses and adaptations. This phenomenon may rely on the presence of a great number of experimentally accredited stress-related motifs and regulatory residues in the active region of these proteins [108,109]. Markedly high transcript levels in response to stresses were detected for members belonging to the *ERF* gene subfamily compared with the *AP2* gene subfamily, and members of *DREB* illustrated high expression in response to heat and drought stresses. These differences in expression may be due to the longer exons and/or lack of introns in *ERF* subfamily genes, which can lead to an extreme reaction and excessive transcription of these genes during plant development and stress exposure, compared to AP2s [81,94]. Members of the *AP2/ERF* gene family have apposite functions, transcription repression, or activation of the downstream genes [53,54]. In the present study, the conserved sequences of three identified repression motifs ([R/K]LFGV, FDLNLPP, and EDLL) were detected into the protein sequence of *TtAP2*s*/ERF*s. The R/KLFGV motif as a repression sequence is distributed in the RAV subfamily [54], while FDLNLPP and EDLL were originally found in the ERF subfamily [53,97]. According to expression profile, most genes that contained a repression motif are not induced by heat and drought stresses in durum wheat, indicating that *TtAP2/ERFs* with activation functions are more involved in response to drought and heat stress. The downregulation of several *TtAP2*s*/ERF*s in this study may play a vital function in the transcriptional adjustment of stress-related genes. These findings indicated that decreased expression of *AP2/ERF*s under stress conditions can help to retain the osmotic potential of plant cell membranes, which ultimately can lead to the accumulation of harmful substances in transport vesicles to inhibit phytotoxic effects [19,95], constituting an initial protection system in dealing with stresses. These results suggest that *TtAP2/ERF* genes may be vastly involved in various developmental systems and abiotic stress responses in durum wheat.

## 5. Conclusions

*AP2/ERF* TFs, which are important regulators of plant growth, development, and stress response, have been studied in depth in several crop species. The present study distinguished and identified 271 members of the *AP2/ERF* gene family in the durum wheat genome, which may facilitate the functional analysis of the other *AP2/ERF* genes in the future. Our bioinformatics analyses and expression profiling provide novel insights into the biological function of these *TtAP2/ERF* genes. Our findings can be effective for elucidating the highly unrecognized *AP2/ERF*-mediated signaling pathways that are critical for the survival of plants under abiotic stress conditions and may provide new opportunities to discover more stress adaptation mechanisms in durum wheat and other plant species.

## Supplementary Materials

The following are available online at https://www.mdpi.com/2073-4425/11/12/1464/s1, Table S1: List of primers used for expression assay of the candidate *AP2/ERF* genes; Table S2: List of the identified *TtAP2/ERF* genes and their characteristics in the durum wheat genome; Table S3: List of the conserved protein motifs predicted in TtAP2/ERF sequences from durum wheat; Table S4: Duplicated gene pairs and the Ka/Ks values predicted in TtAP2/ERF gene family in the durum wheat genome; Figure S1: Phylogenetic relationships of the 271 non-redundant putative TtAP2/ERF proteins predicted from the *T. turgidum* genome according to MEGA software (v. 6.0) based on the neighbor-joining (NJ) method. The 15 conserved motifs have been identified and listed in front of each protein.
