# Supplementary material for: The AP2/ERF Gene Family in Triticum durum: Genome-Wide Identification and Expression Analysis under Drought and Salinity Stresses
Source: Genes (Basel). 2020 Dec 7;11(12):1464. doi: 10.3390/genes11121464 (PMC7762271; doi:10.3390/genes11121464)
Supplement: Supplementary file 1 [file genes-11-01464-s001.zip › Suppl/Table S3.docx]

**Supplementary Table 3 |** List of the conserved protein motifs predicted in TtAP2/ERF sequences from durum wheat

| **Motif No.** | **Sites** | **E-value** | **Amino acid sequence of motif** | **Width**  **(aa)** | **Domain** |
| --- | --- | --- | --- | --- | --- |
| Motif 1 | 265 | 3.3E-38 | KGARVWLGTFDTAEEAARAYDVAA | 24 | AP2 (RAYD element) |
| Motif 2 | 236 | 1.3E-15 | RPWGKWAAEIR | 11 | AP2 (YRG element) |
| Motif 3 | 244 | 9.2E-11 | LRGAAAKLNFP | 11 | C-terminal region of RAYD element |
| Motif 4 | 234 | 6.2E-82 | GRKPRYRGVRQ | 11 | N-terminal region of YRG element |
| Motif 5 | 17 | 2.5E-40 | EAPVMNMYSDQGSNSFGCSDLGWEYDTKTPDISSIAPISTI | 41 | - |
| Motif 6 | 15 | 2.7E-29 | EPWMRFLMDDGVDEPIDSLLNFDVPQDVVGNM | 32 | - |
| Motif 7 | 15 | 9.9E-36 | PSAAQEPSVIPAVNNLANPNAFVYPSADFASNQPLVQPDNVPFVPAMNSV | 50 | - |
| Motif 8 | 10 | 6.1E-35 | LLNFEDGEGKVWRFRYSYWNSSQSYVLTKGWSRFVKEKGLGAGDAVVFYR | 50 | B3 DNA-binding |
| Motif 9 | 17 | 4.0E-23 | HRWTGRYEAHLWDNSCRREGQ | 21 | - |
| Motif 10 | 28 | 6.8E-22 | LAHSLPRPASAAPDDVRAAAALAAAAVR | 28 | AP2 (RAYD element) |
| Motif 11 | 31 | 2.0E-22 | DYEKELEEMKAMSKEEFVASLRRKSSGFS | 29 | - |
| Motif 12 | 28 | 8.3E-20 | VVELEDLGAEYLEELL | 16 | - |
| Motif 13 | 9 | 2.9E-28 | ESRAEAEMLVPQGINMKEITTKKKKTKKPSVVVSAGETDEEAMARFAREH | 50 | - |
| Motif 14 | 10 | 2.0E-25 | PTPAAAREPLFEKTVTPSDVGKLNRLVIPKQHAEKHFPLQLPPATTTTTG | 50 | B3 DNA-binding |
| Motif 15 | 33 | 1.0E-20 | EAPSYYASLAZGMLMEPP | 18 | - |
